# Bactericidal versus bacteriostatic antibacterials: clinical significance, differences and synergistic potential in clinical practice

**DOI:** 10.1093/jac/dkae380

**Published:** 2024-10-29

**Authors:** Angela Ishak, Nikolaos Mazonakis, Nikolaos Spernovasilis, Karolina Akinosoglou, Constantinos Tsioutis

**Affiliations:** Department of Internal Medicine, 48202 Henry Ford Hospital, Detroit, MI, USA; Department of Internal Medicine, Thoracic Diseases General Hospital Sotiria, 11527 Athens, Greece; Department of Infectious Diseases, German Oncology Centre, 4108 Limassol, Cyprus; School of Medicine, University of Crete, 71500 Heraklion, Greece; School of Medicine, University of Patras, 26504 Rio, Greece; Department of Internal Medicine and Infectious Diseases, University General Hospital of Patras, 26504 Rio, Greece; School of Medicine, European University Cyprus, 6 Diogenes str, Nicosia 2404, Cyprus

## Abstract

Antibacterial activity can be classified as either bactericidal or bacteriostatic, using methods such as the MBC/MIC ratio and time–kill curves. However, such categorization has proven challenging in clinical practice, as these definitions only apply under specific laboratory conditions, which may differ from clinical settings. Several factors, such as the specific bacteria or infectious medium, can affect the action of antibiotics, with many antibacterials exerting both activities. These definitions have also led to the belief that bactericidal antibacterials are superior to bacteriostatic, especially in more severe cases, such as endocarditis, neutropenia and bacteraemia. Additionally, current dogma dictates against the combination of bactericidal and bacteriostatic antibacterials in clinical practice, due to potential antagonism. This review aimed to assess the differences in antibacterial activity of bactericidal and bacteriostatic antibacterials based on *in vitro* and *in vivo* studies and examine their antagonistic or synergistic effects. Our findings show that specific bacteriostatic agents, such as linezolid and tigecycline, are clinically non-inferior to bactericidals in multiple infections, including pneumonia, intra-abdominal infections, and skin and soft tissue infections. Studies also support using several bacteriostatic agents as salvage therapies in severe infections, such as neutropenic fever and endocarditis. Additionally, not all combinations of bacteriostatic and bactericidal agents appear to be antagonistic, with many combinations, such as linezolid and rifampicin, already being used. The findings should be interpreted with caution, as most evidence is from observational studies and there is a need for randomized controlled trials to assess their effectiveness and combinations, especially within the context of rising antimicrobial resistance.

## Introduction

The introduction of antimicrobial agents in clinical practice has played a significant role in reducing the morbidity and mortality associated with infections. As antimicrobial discovery rapidly evolved, various antibacterials with diverse mechanisms of action were introduced in clinical use and different classification systems were used to group them according to their function. One classification is based on their mode of action—either bactericidal or bacteriostatic. In simple terms, bacteriostatic antibacterials are defined as those that prevent the growth of the bacteria; bactericidal antibacterials are those that kill bacteria^1^ (Table 1). However, it is crucial to remember that even bacteriostatic antibacterials do kill bacteria, although they do not reach specific laboratory thresholds to be termed as bactericidal.^2^

**Table 1.** Commonly used antibacterials categorized by their activity

Bactericidal agents	Bacteriostatic agents
Aminoglycosides	Tetracyclines
β-Lactams	Macrolides
Fluoroquinolones	Oxazolidinones
Glycopeptides	Sulfonamides
Rifampicin	Lincosamides
Nitroimidazoles	Trimethoprim
Lipopeptides	Chloramphenicol
Polymyxins	Nitrofurantoin
Fosfomycin	Fusidic acid

The MIC is defined as the lowest concentration of the antimicrobial that will inhibit the visible growth of a microorganism at 24 h of growth in specific media, whereas the MBC is the minimum concentration of antimicrobial required to give a 3-log_10_ (≥99.9% killing) reduction in surviving bacteria (cfu/mL) compared with the initial inoculum at 24 h of growth.^3^ To define whether an antibacterial agent is bactericidal or bacteriostatic *in vitro*, the MBC/MIC ratio can be used.^4^ If the MBC/MIC ratio is ≤4, the effect is considered bactericidal, and if the MBC/MIC ratio is >4, the effect is defined as bacteriostatic. There are, however, certain limitations to this definition. Firstly, this definition is laboratory-based, and many of the techniques used to calculate this ratio differ between laboratories; moreover, some studies have used non-standardized methods.^1^ Additionally, these tests are usually done in a specified time frame and against specific bacterial strains, thus providing data on antibacterial agent activity against specific strains only.^1^ Finally, these tests are performed *in vitro* and do not necessarily mimic *in vivo* conditions; they do not consider the host’s immune system capability and its interaction with bacteria and antibacterial agents.

The MIC and MBC of antimicrobials constitute measures of *in vitro* efficacy against bacteria, whereas *in vivo* efficacy and, correspondingly, the optimal antimicrobial dose depend on pharmacokinetic and pharmacodynamic (PK/PD) indices. For concentration-dependent antimicrobials, the ratio of the free maximal concentration to MIC (f*C*_max_/MIC) is critical, whereas time-dependent antimicrobials rely on the time above the MIC expressed as a percentage of the dosing interval (*f*T > MIC). For co-dependent antimicrobials, the ratio of the (unbound) 24 h AUC to MIC (*f*AUC_24_/MIC) is used.^5^ Moreover, calculation of the dose administered is based on body clearance, targeted plasma concentration and drug bioavailability.^5^

Another *in vitro* model that can be used for the characterization of an antibacterial as bactericidal or bacteriostatic is by use of time–kill curves. Even though these curves are not routinely used for this purpose, they can aid in determining the kinetics of bacterial killing and whether it is time-dependent or concentration-dependent.^1^ Time–kill curves are important to understand, as many of the studies discussed in this review use these curves when expanding on antibacterial activity. Typically, they depict that the bacterial concentration stabilizes or is maintained (i.e. growth is halted) when exposed to bacteriostatic agents, whereas there is a steady or rapid reduction in the bacterial concentration when exposed to bactericidal agents.^6–8^

In reality, the activity of antibacterials and whether they will exhibit bactericidal or bacteriostatic *in vitro* activity can be affected by multiple factors, such as the environmental conditions in the medium or tissue, the number of bacteria, the type of bacteria, the duration of the treatment, the dose of the agent and its mechanism of action.^1,9^ Prominent examples of these phenomena are linezolid, eravacycline, ciprofloxacin and some aminoglycosides. Multiple *in vitro* studies have showed that linezolid exhibits bactericidal activity against *Streptococcus* spp. and is bacteriostatic against Gram-positive cocci such as enterococci and MRSA.^10–12^ Eravacylcine is another antibacterial that is generally bacteriostatic against Gram-positive cocci such as staphylococci; however, it has also demonstrated bactericidal activity in *in vitro* studies against certain strains of *Acinetobacter baumannii*, *Escherichia coli* and *Klebsiella pneumoniae*.^13^ Another example is ciprofloxacin, which exhibits bacteriostatic activity when DNA replication is hampered, by inhibiting DNA gyrase, whereas it is bactericidal through bacterial DNA fragmentation.^14^ Table 2 summarizes some of the antibacterials that have demonstrated both bactericidal and bacteriostatic activity in *in vitro* studies.

**Table 2.** Bacteriostatic antibacterial agents with dual bacteriostatic and bactericidal activity

Antibacterial agent	Bactericidal activity	Bacteriostatic activity
Linezolid^10–12^	*Streptococcus pneumoniae*	MRSA
	*Bacteroides fragilis*	VRE
	*Clostridium perfringens*	
Chloramphenicol^15^	*Haemophilus influenzae*	Enterobacterales
	*Streptococcus pneumoniae*	*Staphylococcus aureus*
	*Neisseria meningitidis*	
Eravacycline^13^	*Acinetobacter baumannii*	*Staphylococcus aureus*
	*Escherichia coli*	*Enterococcus faecalis*
	*Klebsiella pneumoniae*	
Tigecycline^16,17^	MRSA^a^	*Enterococcus faecalis*
	MSSA	VRE
	Penicillin-resistant *Streptococcus pneumoniae*	*Escherichia coli*
		*Klebsiella pneumoniae*
		*Streptococcus pneumoniae*

^a^In prolonged therapy.

The aim of this review is to further clarify the terms bactericidal and bacteriostatic, especially in clinical settings, and describe the differences in applications of these agents based on recent data from *in vitro* and *in vivo* studies. Furthermore, this review will examine the antagonistic or synergistic effect of combining bactericidal and bacteriostatic antibacterials.

## Antibacterial combinations

Combination therapy involving different antibacterials has been increasingly used to address the emergence of MDR bacterial infections. However, when choosing combinations it is paramount to understand which antibacterials act synergistically and which antagonistically to optimize treatment strategies. It is generally believed that combinations of bacteriostatic and bactericidal antibacterials should not be used, as *in vitro* studies have shown them to be antagonistic.^18–20^ It is hypothesized that antagonism results because bacteriostatic drugs slow the growth of bacteria, hence reducing the efficiency of bactericidal drugs, as the latter are most potent against actively growing cells.^21^ Antagonism between bactericidal and bacteriostatic agents may also be explained by the balance in synthesis of the different compartments of the bacterial cell (i.e. DNA and proteins), which occurs due to the combination of the agents. More specifically, in the presence of a DNA replication inhibitor (e.g. ciprofloxacin), protein synthesis is not affected and as a result, proteins are produced more rapidly than DNA, leading the bacterial cell to a state of imbalance. When a translation-inhibiting drug (e.g. tetracycline) is added, protein synthesis is suppressed, reinstating the ratio of DNA to protein and rendering bacterial growth faster.^21^ Moreover, some antibiotics (e.g. nitrofurantoin) induce polysaccharide synthesis by bacterial cells. These molecules act as anti-adhesive agents and impede the cellular entry of other drugs (e.g. ciprofloxacin), thereby developing an antagonistic interaction.^22,23^ However, in clinical practice, several such combinations have proved to be synergistic, such as trimethoprim and streptomycin.^21^ An important study that tested this hypothesis was by Ocampo *et al*.,^24^ who combined five different drugs (nalidixic acid, streptomycin, tetracycline, erythromycin and trimethoprim) at clinically relevant inhibitory concentrations and assessed their interaction using time–kill curves; they then extended to assess combinations of 21 different antibiotics at subinhibitory concentrations. Their results confirmed the hypothesis that in the presence of a bacteriostatic agent, the killing rate of bactericidal drugs declined, highlighting that antagonism was present; however, it did differ according to the bactericidal drug used. For example, there were higher degrees of antagonism between nalidixic acid and tetracycline than between trimethoprim and streptomycin.^24^ More importantly, their assessment of the 21 different drug interactions using screening methods revealed more information about antagonism among antibiotics using different mechanisms of action. Their results further proved that antagonism among bactericidal and bacteriostatic drugs was highly prevalent and that specific combinations such as tetracyclines and aminoglycosides with β-lactams, macrolides with fluoroquinolones, and β-lactams with folic acid synthesis inhibitors, frequently showed antagonism.^24^ However, it is important to understand that these results came only from studies on *E. coli* and these combinations could act differently against different bacteria and in *in vivo* environments, where many different factors can affect drug behaviour.

In clinical studies, many combinations of bacteriostatic and bactericidal antibacterials have been used, even though many *in vitro* studies showed antagonistic effects. A landmark study for the treatment of endocarditis was the Partial Oral Treatment of Endocarditis (POET) study that compared partial oral antibiotic regimens with IV antibiotic regimens for the treatment of left-sided endocarditis.^25^ A closer look at the antibiotic combinations used for many cases of bacterial endocarditis revealed that combinations of bacteriostatic and bactericidal antibiotics were frequently used. These combinations included amoxicillin plus fusidic acid, linezolid plus rifampicin, and dicloxacillin plus fusidic acid for *Staphylococcus aureus*, coagulase-negative staphylococci (methicillin-resistant or sensitive) or streptococcal endocarditis.^25^ Furthermore, a linezolid plus moxifloxacin combination was recommended for *Enterococcus faecalis* endocarditis, whereas moxifloxacin and clindamycin were recommended for streptococcal endocarditis.^25^

It appears that linezolid was commonly used in combinations with other bactericidal drugs and is expected to continue to be used, especially against resistant Gram-positive infections. An *in vitro* study assessed the combination of linezolid with other bactericidal drugs using time–kill curves. It showed that the addition of linezolid to gentamicin and vancomycin resulted in reduction of their antibacterial activity and was therefore deemed antagonistic. However, linezolid with rifampicin resulted in an additive interaction against susceptible *S. aureus* strains and inhibited rifampicin-resistant variants.^26^ Another study also revealed that linezolid was synergistic with imipenem, doripenem and plazomicin against MRSA and methicillin-resistant strains of *Staphylococcus epidermidis.*^27^ These studies suggest that not all bacteriostatic/bactericidal combinations are antagonistic, and many can prove synergistic and could aid in reducing resistance. For example, a recent *in vitro* study has examined the combination of linezolid and fosfomycin against 34 clinical isolates of *Enterococcus faecium* and *E. faecalis* and showed both were synergistic, which could aid in suppressing the selection of resistant strains of enterococci against both antibiotics.^28^

Another bacteriostatic agent that has been used in combinations with bactericidal drugs in clinical practice is clindamycin. An interesting combination used is clindamycin/rifampicin; many *in vitro* studies have shown their synergistic activity^29^ and therefore it has been extensively studied in clinical settings in several infections. A 2017 retrospective cohort study in the Netherlands assessed this combination (orally) in treating periprosthetic hip or knee infection by *S. aureus* or coagulase-negative staphylococci in 36 patients following surgical debridement for an early infection or those who underwent aseptic revision of loose components that were later found to be culture-positive.^29^ Their results showed a cure rate of 86%; 78% (14/18) in the surgical debridement group and 94% (17/18) in the revision group.^29^ Another retrospective cohort study in France showed a cure rate of 64.7% by intention to treat and 84.6% by per protocol analysis, using an oral combination of clindamycin/rifampicin in 37 cases of erythromycin-resistant and lincosamide-susceptible bone and joint infections due to *Staphylococcus* spp.^30^ In studies that also assessed this combination in hidradenitis suppurativa, where cases have been associated with *Staphylococcus* spp. infection, the reported success rate for oral clindamycin with rifampicin ranged between 63.6% and 85.7%.^31^

Tigecycline with colistin is another combination that has been extensively studied, especially against resistant Enterobacteriaceae. A 2012 *in vitro* study assessed the interaction between tigecycline and colistin against eight NDM-1-producing Enterobacteriaceae using time–kill curves, and showed that the addition of tigecycline to colistin sulphate and colistin methanesulfonate did not increase bacterial killing and in low concentration was antagonistic.^32^ However, a more recent *in vitro* study showed that this combination was effective against five carbapenem-resistant NDM-producing *E. coli* strains, with colistin allowing increased uptake of tigecycline.^33^ These results were reproducible in an *in vitro* study that evaluated this combination against MDR *A. baumannii*, with tigecycline increasing the bactericidal effects of colistin.^34^ There is also a limited number of studies that assessed this combination in clinical settings. A retrospective cohort study from Thailand assessed the use of IV colistin and tigecycline compared with IV tigecycline monotherapy in the treatment of 28 patients with post-surgical non-bacteraemic intra-abdominal infections (IAIs) due to carbapenem-resistant *A. baumannii* (CRAB), and showed that 14 day, 30 day and in-hospital mortality rates, the rate of breakthrough bacteraemia and the rate of bacterial eradication, were not significantly different between the two groups, with the combination therapy associated with more severe renal impairment and higher costs.^35^ Additionally, a larger cohort study from Spain with 118 patients with bacteraemia due to CRAB showed similar results, where the 30 day crude mortality was not significantly different in the combination therapy as compared with colistin monotherapy.^36^ These results highlight that, even though the majority of *in vitro* studies demonstrated synergy among both antibiotics, this does not necessarily translate to increased effectiveness in clinical settings, as patient factors such as severity of illness and comorbidities also play an important role in response to treatment.

## Head-to-head comparison of bactericidal versus bacteriostatic antibacterials in common infections

### Pneumonia

Bacterial pneumonia remains a significant cause of mortality, especially in older patients with comorbidities.^37^ Current guidelines on management of community-acquired pneumonia (CAP), hospital-acquired pneumonia (HAP) and ventilator-associated pneumonia (VAP), offer all necessary information to healthcare professionals.^38,39^ To this end, a head-to-head comparison between bactericidal and bacteriostatic antibacterials in the treatment of bacterial pneumonia is of interest.

In a study performed nearly 30 years ago in the Netherlands, the effect of oral azithromycin was compared with IV benzylpenicillin in 104 hospitalized patients with suspected pneumococcal CAP, demonstrating higher clinical and radiological success rates with azithromycin, although statistical significance was not reached.^40^ Two years later, an open prospective randomized study contrasted IV clarithromycin with amoxicillin/clavulanic acid in 112 hospitalized patients with CAP in Switzerland. The rate of clinical improvement was similar for the two administered drugs.^41^ In a subsequent study azithromycin was compared with cefuroxime in the treatment of 180 patients with CAP, demonstrating similar clinical efficacy and a shorter duration of therapy.^42^

In the context of antibacterial management of CAP, direct comparison of tigecycline with levofloxacin has been a matter of interest in multiple studies, indicating non-inferiority of tigecycline and revealing comparable cure rates for the two antimicrobial agents.^43–45^ Furthermore, doxycycline, another tetracycline derivative, was studied side by side with levofloxacin in the treatment of patients with CAP requiring hospitalization in a teaching hospital in Cleveland, Ohio.^46^ According to the results of this prospective double-blinded trial the therapeutic efficacy was not significantly different between the two drugs.^46^ More recently, omadacycline, a newer tetracycline, has been approved in the USA for the treatment of CAP, based on the results of the Omadacycline for Pneumonia Treatment in the Community (OPTIC) trial, an international clinical trial that contrasted this agent with moxifloxacin for treatment of hospitalized patients with CAP, indicating similar clinical response rates for the two drugs.^47^ This agent constitutes an effective oral alternative option to fluoroquinolones in patients with CAP and pneumonia severity index (PSI) risk class II/III and at least one comorbidity.^48^ Additional subgroup analyses of the OPTIC study based on disease severity risk scores and presence of COPD/asthma or bacteraemia, demonstrated that omadacycline was inferior to moxifloxacin only in the context of bacteraemia, exhibiting lower success rates at early clinical response.^49^

Finally, with regard to aspiration pneumonia in elderly patients, IV clindamycin as monotherapy was compared with ampicillin/sulbactam and panipenem/betamiprom during a randomized prospective study in Japan. Researchers observed that clindamycin was cost-effective and had results similar to the two other agents regarding cure and adverse event rates, concluding that clindamycin monotherapy is an effective therapeutic option against aspiration pneumonia.^50^

With respect to nosocomial pneumonia, a multicentre, randomized, double-blind study was carried out at 138 sites in 31 countries, comparing tigecycline with imipenem/cilastatin in 945 patients with HAP. Patients received either tigecycline (100 mg daily) plus an optional adjunctive therapy with ceftazidime for *Pseudomonas aeruginosa* coverage (and addition of an aminoglycoside in some cases for double coverage) or imipenem/cilastatin plus an optional adjunctive treatment with vancomycin for MRSA coverage. Overall mortality was similar between the two groups of patients; however, death rates were higher in the subgroup of VAP patients treated with tigecycline.^51^ Nevertheless, in a following study investigating the same topic, researchers used higher doses of tigecycline and observed a numerically higher clinical response with the use of 200 mg of tigecycline daily.^52^

Direct comparison of linezolid with vancomycin in the treatment of suspected MRSA nosocomial pneumonia has also grabbed the attention of researchers. A systematic review and meta-analysis including nine randomized controlled trials (RCTs), compared linezolid with vancomycin in the treatment of 2618 patients with suspected MRSA pneumonia, and showed that the overall clinical cure rate, the clinical cure rate for patients with culture-confirmed MRSA pneumonia, and the MRSA eradication rate were similar for the two antimicrobials.^53^ A subsequent systematic review and comparative meta-analysis including seven RCTs with 1239 patients and eight retrospective cohort or case-control studies with 6125 patients, analysed the effect of linezolid versus vancomycin against proven MRSA pneumonia. The clinical cure rate and the microbiological eradication rate were significantly lower in the vancomycin-treated subgroup of patients in the RCTs. Mortality rates were indistinguishable for the two treatments in both RCTs and cohort or case-control studies.^54^ These results may reflect the better accessibility of linezolid compared with vancomycin in the epithelial lining fluid, rendering this agent an effective treatment against MRSA pneumonia.

Overall, a systematic review and meta-analysis, published in 2015 by Nemeth *et al.*,^55^ included 33 RCTs and compared bacteriostatic with bactericidal antibiotics in the management of severe infections. Subgroup analysis of 13 studies that covered pneumonia showed no significant difference in clinical cure, mortality and relapse rates between patients treated with bacteriostatic or bactericidal antibiotics. These results were compatible with the findings of a subsequent systematic literature review by Wald-Dickler *et al.*,^2^ which also investigated bactericidal versus bacteriostatic antibiotics in the treatment of bacterial infections, including 56 RCTs. In 19 trials on pneumonia and additional trials that included pneumonia in specific subgroup analyses, non-inferiority of bacteriostatic agents regarding clinical efficacy was observed, except for the aforementioned study of tigecycline versus imipenem/cilastatin in HAP patients, which demonstrated inferiority of tigecycline in patients with VAP.^51^ Finally, a systematic review and meta-analysis of 43 RCTs contrasting bactericidal with bacteriostatic antibiotics in the treatment of pneumonia indicated no statistically significant difference with respect to clinical cure, mortality, microbiological eradication and treatment failure rates between the subgroups of patients treated either with bactericidal or with bacteriostatic agents. This meta-analysis included RCTs with patients having CAP and HAP, and trials that compared multiple categories of antibacterials, such as oxazolidinones with glycopeptides, macrolides with fluoroquinolones, and macrolides with β-lactam antibacterials.^56^

### Intra-abdominal infections

Tigecycline, a broad-spectrum glycylcycline, has been used for the treatment of complicated intra-abdominal infections (cIAIs) because of its activity against multiple microorganisms, such as Enterobacteriaceae, *E. faecalis*, *E. faecium* and anaerobes, constituting a suitable antibacterial option in polymicrobial infections.^57^ A double-blind, multinational study comparing tigecycline with imipenem/cilastatin for treating patients with cIAIs exhibited similar clinical cure rates.^58^

Two randomized studies of patients with cIAIs comparing tigecycline with a combination regimen of ceftriaxone plus metronidazole have shown non-inferiority of tigecycline.^59,60^ The first study was conducted in the USA, Canada and Latin America, evaluated clinically 376 patients, and exhibited similar clinical response rates between the two treatment arms.^59^ The second study was an international trial that evaluated 387 patients clinically and 227 patients microbiologically. Similar clinical and microbiological response rates were revealed between tigecycline and comparators.^60^

In the age of MDR, eravacycline, a newer tetracycline derivative, represents an additional weapon in the therapeutic arsenal against resistant bacteria, even CRAB.^61^ This agent has been recently approved for use in the treatment of cIAIs, as it demonstrated non-inferiority compared with ertapenem and meropenem in cIAIs in two double-blind clinical trials, IGNITE (the Investigating Gram-Negative Infections Treated With Eravacycline) trial 1 and IGNITE4.^62,63^ In terms of real-world evidence, according to the results from a Bayesian network meta-analysis including 25 RCTs and 9372 adult patients, it was indicated that eravacycline had similar clinical efficacy and safety in patients with cIAIs, compared with common bactericidal antibacterials, such as meropenem, ertapenem, ceftazidime/avivactam plus metronidazole, piperacillin/tazobactam, imipenem/cilastatin, and ceftriaxone plus meronidazole. Moreover, eravacycline proved significantly better than tigecycline with respect to microbiological response rates.^64^

### Skin and soft tissue infections

Skin and soft tissue infections (SSTIs) represent a spectrum of microbial invasion involving the epidermis, dermis and underlying soft tissues, and are among the most frequent bacterial infections. Constituting a leading cause for antimicrobial prescriptions, SSTIs contribute to approximately 10% of hospital admissions in the USA.^65,66^ SSTIs can range from erysipelas and common cellulitis to more serious conditions, such as necrotizing fasciitis or abscesses, which warrant meticulous wound care and surgical interventions in severe cases.^66^

In 2014, the IDSA released guidelines outlining the treatment of SSTIs.^67^ In brief, management of SSTIs is dependent on the severity, nature and type of infection. Purulent SSTIs, whether mild, moderate or severe, are recommended to be treated with incision and drainage followed by empirical treatment with one of vancomycin, daptomycin, linezolid, telavancin or ceftaroline in severe, purulent infections.^68^ Moderate purulent infections, following incision and drainage, are empirically treated with trimethoprim/sulfamethoxazole or doxycycline.^68^ Severe, non-purulent SSTIs are managed with aggressive surgical debridement to remove any necrotizing processes and empirically treated with vancomycin plus piperacillin/tazobactam.^68^ Moderate non-purulent SSTIs are managed with IV penicillin, ceftriaxone, cefazolin or clindamycin.^68^ Finally, mild and non-purulent SSTIs are treated with oral penicillin, cephalosporin, dicloxacillin or clindamycin.^68^

Numerous studies have compared the efficacy of linezolid, a bacteriostatic, with vancomycin, a bactericidal, as monotherapy. Itani *et al*.^69^ conducted an open-label multicentre study in the USA and found linezolid to be statistically superior to vancomycin in the treatment of complicated SSTIs caused by MRSA, revealing that hospitalized patients with linezolid had a significantly shorter length of stay and duration of IV therapy. Several other studies comparing the two antibiotics found no statistical superiority in clinical outcomes and microbiological eradication in hospitalized patients with MRSA and other complicated SSTIs involving Gram-positives.^70,71^ Other RCTs from Taiwan^72^ and India^73^ have compared tigecycline, a bacteriostatic, with vancomycin alone or with aztreonam in complicated SSTIs in hospitalized patients, and revealed that tigecycline monotherapy is statistically non-inferior to the combination of vancomycin and aztreonam; however, tigecycline use was associated with higher rates of nausea, dyspepsia and anorexia, whereas vancomycin and aztreonam combination led to pruritus and rash.^72,73^ In 2007 Cenizal *et al.*^74^ reported a prospective randomized trial in the USA, comparing the efficacy of doxycycline monotherapy with trimethoprim/sulfamethoxazole for outpatient treatment of SSTIs and revealed no statistical difference in clinical outcomes. Similarly, linezolid monotherapy compared with teicoplanin monotherapy, trimethoprim/sulfamethoxazole combined with rifampicin, or dalbavancin monotherapy, showed no statistical difference of clinical outcomes in treatment of MRSA SSTIs in hospitalized patients.^75–77^ A meta-analysis conducted by Nemeth *et al*.^55^ revealed that linezolid demonstrated statistically significantly higher clinical cure rates when compared with its bactericidal comparators and reported near-significant association of increased mortality in patients treated with tigecycline.

### Osteomyelitis and prosthetic joint infections

Linezolid constitutes a reasonable antimicrobial therapeutic choice for bone infections, when a Gram-positive microorganism is the causative factor, including MRSA. The oral bioavailability of linezolid and its sufficient concentrations inside bone tissue render this agent an attractive option against this type of infection, which requires prolonged treatment.^78^ Limited data exist regarding direct comparison of linezolid with bactericidal antibacterials in the treatment of bone infections. In a case-control study in Greece, linezolid was administered to 34 patients with osteomyelitis or prosthetic joint infections (PJIs), and results concerning efficacy and safety were compared with a group of well-matched controls, who received monotherapy or combination regimen, including at least one bactericidal agent.^78^ Although initial treatment success rates were similar for the two subgroups, relapse rates were higher, and discontinuation of antimicrobial therapy was more prevalent in the linezolid group. Poor tolerability occurred mostly due to myelosuppression.^78^ Moreover, a retrospective study in China compared linezolid and vancomycin regimens combined with one-stage surgery, followed by implantation of a vancomycin-loaded calcium sulphate artificial bone in 64 patients with traumatic osteomyelitis of the limbs caused by MRSA. This showed that both antimicrobials had satisfactory clinical outcomes, yet linezolid-treated patients had fewer adverse events, a shorter duration of antimicrobial therapy and a shorter hospital stay.^79^ Regarding PJIs, a retrospective cohort study in Japan compared linezolid with daptomycin in 82 patients with PJIs caused by Gram-positive microorganisms, revealing comparable success rates for the two drugs. Nonetheless, lower c-reactive protein (CRP) values and fewer adverse events were observed in the daptomycin-treated subset of patients.^80^ Finally, in a randomized non-inferiority trial in Switzerland of osteoarticular infections comparing linezolid monotherapy versus trimethoprim/sulfamethoxazole combined with rifampicin against various types of MRSA infections, higher relapse rates were observed in the linezolid group. Nevertheless, a major limitation of this trial was the small sample size.^75^

Conclusively, linezolid, a bacteriostatic agent, is a valuable therapeutic option in bone infections caused by Gram-positive microorganisms, including MRSA, especially in terms of treatment completion in outpatient settings, because of its high oral bioavailability. However, caution is required in detecting adverse effects, such as haematological toxicity, peripheral neuropathy and lactic acidosis, during prolonged treatment courses. Tedizolid, a more recent oxazolidinone, may feature a more favourable safety profile than linezolid in case of lengthy treatment of osteoarticular infections,^81^ yet clinical data are still scarce.

Clindamycin also constitutes a bacteriostatic agent that can be used for the treatment of Gram-positive bone infections.^30^ However, data are lacking regarding head-to-head comparisons of this agent with bactericidal drugs. It is usually used in combination with other bactericidal antibacterials, such as rifampicin, fusidic acid or trimethoprim/sulfamethoxazole.^30^

### Other infections

For treatment of enteric (typhoid and paratyphoid) fever, a disease caused by *Salmonella enterica* serotype Typhi and *S. enterica* serotype Paratyphi, bacteriostatic antibacterials have been compared with bactericidal antibacterials in several studies. Azithromycin has been compared with third-generation cephalosporins, such as ceftriaxone and cefixime, exhibiting non-inferiority regarding clinical outcomes in children with uncomplicated typhoid fever.^82,83^ Moreover, a randomized trial conducted in Egypt more than 20 years ago revealed that azithromycin was clinically and microbiologically equally effective to ciprofloxacin, when administered for a 7 day treatment course of adult patients with uncomplicated disease.^84^

Another bacteriostatic agent, chloramphenicol, was compared with ceftriaxone in a randomized trial for treatment of 46 adults and children who had positive blood cultures for *S. enterica* serotype Typhi or *S. enterica* serotype Paratyphi, demonstrating similar cure rates.^85^ Chloramphenicol has also been compared with fluoroquinolones, such as ciprofloxacin and gatifloxacin, for the treatment of enteric fever, revealing equivalent clinical efficacy, although requiring longer treatment duration.^86^

Despite these findings, the decision about the optimal antimicrobial option should not be based on the bacteriostatic or bactericidal action of the agent. It should be guided by the regional rates of antimicrobial resistance, as MDR and XDR *S. enterica* serotype Typhi isolates are increasing worldwide, rendering the understanding of patterns and trends of antimicrobial resistance essential to avoid treatment failure.^87^

With respect to plague, a disease considered extinct in Europe, anecdotal evidence exists regarding direct comparisons of bactericidal with bacteriostatic antimicrobials in the treatment of this infectious disease. Plague is caused by *Yersinia pestis*, remains a public health concern in countries of sub-Saharan Africa, Asia and the Americas, and can be treated with various classes of antibacterials, such as fluoroquinolones, aminoglycosides, tetracyclines and chloramphenicol.^88^ In a randomized clinical trial in Tanzania, 65 adult or paediatric patients with bubonic, septicaemic or pneumonic plague were enrolled, who received treatment with either gentamicin or doxycycline for 7 days. Results were similar with high success rates for both regimens.^89^

## Role of bacteriostatic antibacterials compared with bactericidal antibacterials in severe infections

It is a long-held strategy in treatment of infections such as endocarditis, meningitis, bacteraemia or infections in immunocompromised patients including febrile neutropenia, to use a bactericidal antibacterial in order to increase the odds of cure. However, with the rise of resistance to several bactericidal agents, use of bacteriostatic agents could prove useful, if not imperative. In this section, the recent literature evaluating the use of bacteriostatic agents in severe infections is summarized. Table 3 summarizes the studies that have assessed bacteriostatic antibacterials in patients with neutropenic fever, endocarditis, Gram-positive bacteraemia and meningitis.

**Table 3.** Summary of the characteristics and results of studies that have assessed the use of bacteriostatic antibacterials in severe infections such as febrile neutropenia, meningitis, bacteraemia and infective endocarditis

Author (year)	Type of study	Patient characteristics	Disease	Bacteriostatic antibiotic (number of patients)	Bactericidal antibiotic (number of patients)	Main pathogen	Main results
Bucaneve *et al.* 2004	RCT (open label)	Hospitalized adults with neutropenic fever secondary to haematological malignancy	Neutropenic fever	Tigecycline + piperacillin/tazobactam (*n* = 187)	Piperacillin/tazobactam (*n* = 205)	Multiple (Gram-negatives and Gram-positives including MRSA)	Clinical success rate was 67.9% of patients in the combination group, compared with 44.3% of patients in the monotherapy group (*P* < 0.001). The combination regimen had a higher clinical success than monotherapy in bacteraemia (0.5%, compared with 27.7%; *P* < 0.001)
Jaksic *et al.* 2006	RCT (double-blind)	Hospitalized adults with neutropenic fever secondary to malignancy	Neutropenic fever	Linezolid (*n* = 304)	Vancomycin (*n* = 301)	Gram-positive including MRSA	No statistically significant difference in clinical success [linezolid group 219 (87.3%) of 251; vancomycin group 202 (85.2%) of 237]; microbiological success rates [linezolid group, 41 (58%) of 71 patients; vancomycin group, 29 (50%) of 58 patients]; and mortality rate [linezolid group, 17 (5.6%) of 304; vancomycin group 23 (7.6%) of 301 vancomycin-treated patients]. Drug-related adverse events (24.0% vs 17.2%; *P* = 0.04) and renal failure (2.3% vs 0.3%; *P* = 0.04) were higher in the vancomycin group. No difference in haematological adverse effects
Faella *et al.* (2006)	Case series	Patients with pneumococcal meningitis	Meningitis	Linezolid combined with ceftriaxone (*n* = 7)	N/A	Penicillin-resistant *Streptococcus pneumoniae*	5/7 patients treated with the combination of ceftriaxone and linezolid survived
Munoz *et al.* (2007)	Case series	Adult patients with Gram-positive endocarditis and refractory disease, intolerance to other drugs or need for oral consolidation treatment	Gram-positive endocarditis	Linezolid (*n* = 9), in combination with rifampicin in one case	N/A	*Staphylococcus aureus* (including MRSA; *n* = 6), *Streptococcus mutans* (*n* = 1), *Corynebacterium striatum* (*n* = 1), coagulase-negative staphylococci (*n* = 1)	Clinical and microbiological cure with no adverse effects or relapses for all 9 cases
Lauridsen *et al.* (2012)	Retrospective cohort study	Patients with infective endocarditis that failed first-line treatment	Infective endocarditis	Linezolid (*n* = 38)	N/A	Gram-positive cocci (including some cases of culture-negative endocarditis)	No statistically significant differences in cure rate (74% vs 71%, *P* > 0.05), in-hospital mortality (13% vs 14%, *P* > 0.05) or post-discharge mortality at 1 year follow-up (26% vs 26%, *P* > 0.05) for patients receiving treatment with linezolid compared with patients without such treatment
Sipahi *et al.* (2013)	Case series	Patients with MRSA meningitis	Meningitis	Linezolid (*n* = 9)	Vancomycin (*n* = 8)	MRSA	Patients’ treatment with linezolid had a greater rate of MRSA clearance from CSF on Day 5 (7/9 vs 2/8; *P* = 0.044). One-month survival of the patients with microbiological cure was 2/2 in the vancomycin group and 4/7 in the linezolid group
Balli *et al.* (2014)	Systematic review and meta-analysis (including 10 retrospective studies)	Adult patients with VRE bacteraemia	VRE bacteraemia	Linezolid (*n* = 538)	Daptomycin (*n* = 429), heterogeneity in daptomycin dosage	VRE	Higher 30 day all-cause mortality (OR, 1.61; 95% CI, 1.08–2.40) and infection-related mortality (OR, 3.61; 95% CI, 1.42–9.20) for patients treated with daptomycin. Overall mortality was also significantly increased among patients treated with daptomycin (OR, 1.41; 95% CI, 1.06–1.89). Relapse rates were higher in the daptomycin group of patients (not statistically significant), and adverse events did not demonstrate a statistically significant difference between the two subgroups of patients
Chuang *et al.* (2014)	Systematic review and meta-analysis (including 13 retrospective studies)	Patients with VRE bacteraemia	VRE bacteraemia	Linezolid (*n* = 656)	Daptomycin (*n* = 532), heterogeneity in daptomycin dosage	VRE	Mortality was higher in patients receiving daptomycin (OR, 1.43; 95% CI, 1.09–1.86; *P* = 0.009) and subgroup analysis of studies that reported adjusted ORs indicated that daptomycin was associated with higher mortality (OR, 1.59; 95% CI, 1.02–2.50; *P* = 0.04)
Zhao *et al.* (2016)	Systematic review and meta-analysis (including 11 retrospective studies)	Patients with VRE bacteraemia	VRE bacteraemia	Linezolid (*n* = 894)	Daptomycin (*n* = 445), standard or high dose	VRE	Similar overall crude mortality (RR = 1.07; 95% CI, 0.83–1.37), clinical cure (RR = 1.11; 95% CI, 0.88–1.42), microbiological cure (RR = 0.99; 95% CI, 0.90–1.09) and relapse (RR = 1.08; 95% CI, 0.76–1.52) rates between daptomycin- and linezolid-treated patients. No statistically significant difference regarding adverse events between the two groups
Zhou *et al.* (2018)	Prospective observational study	Hospitalized adults with neutropenic fever that failed first-line treatment	Neutropenic fever	Tigecycline (*n* = 125)	N/A	Gram-negative organisms	Clinical success rate was 68.0% (85/125) and mortality rate was 18% (23/125). Clinical success rate in patients with pneumonia as cause of neutropenic fever was 73.1% (49/67). Clinical success in patients with bacteraemia was 35.3% (6/17), with the 30 day mortality rate of 64.7% (11/17)
Sipahi *et al.* (2018)	Case series	Patient with post-neurosurgical meningitis	Meningitis	Linezolid (*n* = 17)	N/A	Methicillin-resistant staphylococcal spp.	15/17 patients achieved microbiological cure with linezolid and 1 reported death due to treatment failure without documented relapse of meningitis due to methicillin-resistant *Staphylococcus* spp.
Shi *et al.* (2019)	Systematic review and meta-analysis (including 21 retrospective observational studies and 1 prospective cohort study)	Patients with VRE bacteraemia	VRE bacteraemia	Linezolid (*n* = 2053)	Daptomycin (*n* = 1934), heterogeneity in daptomycin dosage	VRE	There was a trend towards increased mortality for those in the daptomycin arm vs those in the linezolid arm, although this trend did not reach statistical significance (OR, 1.27; 95% CI, 0.99–1.63; *I*^2^ = 42.9%). In the subset of studies focusing on high-dose daptomycin, comparable mortality associated with daptomycin and linezolid treatment was observed (OR, 0.92; 95% CI, 0.46–1.84; *I*^2^ = 49.4%). Overall, clinical response, microbiological cure, recurrence of bacteraemia and risk of CPK elevation were similar for the two agents. Finally, risk of thrombocytopenia was significantly lower in the daptomycin group of patients
Munoz *et al.* (2021)	Retrospective cohort study	Patients with infective endocarditis treated with linezolid	Infective endocarditis	Linezolid (*n* = 295), only 11 patients were included for comparison with patients not treated with linezolid	N/A	*S. aureus*, coagulase-negative staphylococci, *Streptococcus* spp.	Patients treated with linezolid presented higher in-hospital mortality in contrast to matched controls not treated with linezolid (54.5% vs 18.2%, *P* = 0.04)
Modemann *et al.* (2022)	Retrospective observational study	Hospitalized adults with neutropenic fever with unknown origin secondary to AML/ALL	Neutropenic fever	Tigecycline ± carbapenem, vancomycin or linezolid (*n* = 43)	Carbapenem, vancomycin or linezolid (*n* = 30)	MDR pathogens (including MRSA and VRE)	No statistically significant difference in response rate; however, in patients receiving tigecycline there was lower absolute sepsis (33% vs 47%, *P* = 0.235) and infection-associated mortality rates (5% vs 13%, *P* = 0.221)
Kawasuji *et al.* (2023)	Systematic review and meta-analysis [including 2 RCTs, 1 pooled analysis of 5 RCTs, 1 subgroup analysis (1 RCT) and 5 case-control and cohort studies]	Patients with MRSA bacteraemia	MRSA bacteraemia	Linezolid (*n* = 293)	Daptomycin (*n* = 114), vancomycin (*n* = 4894), teicoplanin (*n* = 27)	MRSA	All-cause mortality, clinical and microbiological cure, hospital length of stay, recurrence, 90 day readmission and adverse event rates were similar between patients treated with linezolid vs those treated with vancomycin, teicoplanin or daptomycin

ALL, acute lymphocytic leukaemia; AML, acute myeloid leukaemia; CPK, creatine kinase; RR, relative risk.

### Neutropenia

A 2009 systematic review of the role of linezolid in infections caused by Gram-positives, including VRE in neutropenic patients, included five studies (two of which were prospective comparative studies) and eight case studies where linezolid was used on a compassionate basis (i.e. after all available treatment options failed) in 438 neutropenic patients.^90^ The overall cure rate ranged from 57% to 87%. Interestingly, in the prospective studies there was a 100% microbiological cure rate against VRE. Moreover, only 0.02% of bacterial isolates developed resistance to linezolid.^90^ Of note, one of the included trials (a double-blinded, prospective RCT) compared vancomycin with linezolid in approximately 600 neutropenic patients and showed no statistically significant difference in clinical success rates (linezolid, 87.3%; vancomycin, 85.2%; *P* = 0.52) and mortality rates (linezolid, 5.6%; vancomycin 7.6%).^91^ Moreover, linezolid was associated with fewer drug-related adverse events and drug-induced renal failure.^91^ One of the concerns of using linezolid in neutropenic patients for Gram-positive coverage is the reported myelosuppression when used for longer than 14 days. Interestingly though, the above-mentioned trial showed that blood count recovery was comparable among patients who received vancomycin and those who received linezolid.^91^

Another bacteriostatic agent studied in neutropenic patients is tigecycline. An open-label trial in 2014 from Italy compared monotherapy with piperacillin/tazobactam (a bactericidal agent) with a combination of piperacillin/tazobactam plus tigecycline (a bacteriostatic agent) in 300 cancer patients with febrile neutropenia.^92^ The intention-to-treat analysis showed that there was a 23.6% risk difference in favour of combination therapy in achieving a clinical cure. This was significant for neutropenic patients with bacteraemia and clinically documented infections.^92^ Another prospective cohort study from China assessed the use of salvage tigecycline (i.e. following treatment failure with first-line therapies) in 125 neutropenic patients with haematological malignancies and showed a 68.0% treatment success.^93^ In this study, tigecycline was not compared with other agents; however, it was used after bactericidal combination therapies such as carbapenems with vancomycin had failed.^93^ The use of another antibacterial in combination with tigecycline was not restricted and, of note, the most common combinations were with bactericidal agents, such as carbapenems and cephalosporins.^93^ In both studies mentioned above, the majority of isolated bacteria were those that tigecycline is known to have bacteriostatic activity against, such as VRE, *K. pneumoniae* and other Gram-negative bacteria.^92,93^ A more recent retrospective single-centre study conducted in 2022 in Germany assessed tigecycline as salvage therapy in 73 neutropenic patients with leukaemia, either as monotherapy (*n* = 30) or in combination with a carbapenem (*n* = 43). Response rates and mortality rates were similar among both groups.^94^ It is important to note that in studies where combination therapies were used, the role of the bactericidal drug was to provide coverage against *Pseudomonas* spp.^93,94^ Overall, these studies show that bacteriostatic agents can be effective and safe in neutropenic patients and their combination with bactericidal agents to broaden antimicrobial coverage is possible and can achieve significant clinical improvement.

### Infective endocarditis

It is widely believed that bacteriostatic agents are ineffective in the treatment of infective endocarditis, and that bactericidal antibacterials are the management hallmark. This is partly due to older studies that have shown poor outcomes when using bacteriostatic agents in endocarditis. Additionally, due to the large concentration of bacteria that can grow on cardiac valves and the poor accessibility of phagocytic cells, it is accepted that non-phagocytic killing by bactericidal agents is crucial to achieving a microbicidal cure.^1,2^ There are no current clinical trials that compare the effectiveness of specific bacteriostatic agents with bactericidal agents in treating endocarditis. However, due to the increase in drug-resistant cases and the development of newer bacteriostatic agents that can reach desirable levels in the bloodstream, there have been some published studies that assess bacteriostatic agents in endocarditis.

One example is linezolid, which was also regarded by the POET study as optimal for oral consolidation in endocarditis in combination with bactericidal drugs. A case series from 2007 described nine patients who were diagnosed with Gram-positive endocarditis and were treated with linezolid due to failure of previous therapies or due to allergies to first-line therapies. All nine patients achieved clinical and microbiological cure with no adverse effects or relapses.^95^ Additionally, several published case reports document the successful treatment of endocarditis caused by MRSA, *Corynebacterium* spp. and VRE, using linezolid.^96–100^ Interestingly, in two cases, linezolid was combined with bactericidal drugs, such as daptomycin and ciprofloxacin.^98,99^ In all these cases, linezolid was used either due to vancomycin resistance, gentamicin resistance or failure of other first-line treatments to achieve microbiological cure.

The largest study to date describing the use of linezolid in endocarditis is a 2021 retrospective cohort study from Spain that compared in-hospital and 1 year mortality among patients who received linezolid for endocarditis treatment (*n* = 295) versus patients who did not (*n* = 3172).^101^ When the authors were trying to assess the true impact of linezolid, only 11 cases fulfilled the criteria and were considered to have been administered linezolid as a definite treatment for endocarditis. Thus, only these 11 cases were used for comparison. Their results showed that patients treated with linezolid had higher in-hospital mortality (54.5% versus 18.2%, *P* = 0.04); however, it is important to note that patients receiving linezolid were affected by a larger number of comorbidities and endocarditis complications, which could have affected the mortality rate.^101^ Another retrospective study from Denmark also compared linezolid (*n* = 38) with other treatments (*n* = 512) in left-sided Gram-positive endocarditis. Their results showed that there were no significant differences in the cure rate, in-hospital mortality or mortality at 12 months between linezolid and other treatments.^102^ These studies show that linezolid could prove to be an effective and well-tolerated treatment for endocarditis caused by Gram-positive organisms, especially MRSA and VRE; however, larger studies are needed to draw more concrete conclusions.

### VRE and MRSA bacteraemia

The dogma that rapid killing of bacteria in bloodstream infections, by use of antibacterials traditionally considered bactericidal, is also not clinically relevant in the case of linezolid use in bacteraemia caused by resistant Gram-positive microorganisms. In a systematic review and meta-analysis published in 2014 including 10 retrospective studies and 967 patients with VRE bacteraemia, treatment with linezolid compared with daptomycin exhibited lower 30 day all-cause, infection-related and overall mortality, as well as comparable adverse event rates.^103^ These results were compatible with mortality outcomes presented in another meta-analysis, which included 13 retrospective cohort studies and 1188 patients with VRE bacteraemia, and compared linezolid with daptomycin, showing higher mortality rates in the daptomycin subgroup.^104^ However, it is of utmost importance to emphasize the heterogeneity in daptomycin dosage used in the above-mentioned studies. In several of them, daptomycin dosage was approximately 6 mg/kg/day, whereas some studies did not mention dosage.^103,104^ Bactericidal activity of daptomycin is dose-dependent, and the safety and efficacy of high-dose daptomycin (>6 mg/kg/day) in difficult-to-treat Gram-positive infections have been well established.^105^

Another meta-analysis published by Zhao *et al.*^106^ in 2016, included 11 retrospective cohort studies and 1339 patients, and compared the efficacy of linezolid versus standard- or high-dose daptomycin in VRE bacteraemia, observing similar overall mortality, clinical cure, microbiological cure, relapse and adverse event rates between the two agents. More recently, Shi *et al.*^107^ conducted a meta-analysis on the same topic and compared the above-mentioned drugs in treatment of VRE bacteraemia. The researchers included 22 observational studies involving 3987 patients and found a non-significant higher mortality rate and a significantly lower risk of thrombocytopenia in patients treated with daptomycin.^107^ In the daptomycin-treated subgroup, a trend towards higher clinical response and microbiological cure as well as lower risk for bacteraemia relapse was observed, though without statistical significance. Moreover, a subgroup analysis of studies using high-dose daptomycin revealed similar mortality rates between the two drugs.^107^

The above-mentioned results should be interpreted with caution, as most of these studies are observational with high heterogeneity in various parameters. The design of larger randomized trials is essential to shed light on the comparison of linezolid versus daptomycin in VRE bacteraemia. Nevertheless, these data rendered linezolid, traditionally considered a bacteriostatic drug, a protagonist in the treatment of this specific bloodstream infection.

The favourable pharmacological properties of linezolid have led to its use in MRSA bacteraemia. A recent systematic review and meta-analysis included two RCTs, one pooled analysis of five RCTs, one subgroup analysis (one RCT), and five case-control and cohort studies involving 5328 patients and compared the efficacy and safety of linezolid versus vancomycin, teicoplanin or daptomycin, in patients with MRSA bloodstream infection. The primary effectiveness outcome was all-cause mortality, and secondary effectiveness outcomes were clinical and microbiological cure, length of hospital stay, recurrence and 90 day readmission rates. The primary safety outcome was defined as the rate of drug-related adverse events. Despite several limitations in the meta-analysis, all the above-mentioned outcomes were similar between patient subgroups, concluding that linezolid could constitute a first-line therapeutic option in MRSA bacteraemia.^108^ Owing to its oral form, linezolid remains a useful therapeutic option in MRSA bacteraemia, with studies showing similar clinical success rate with treatment completion compared with parenteral antibiotics in selected low-risk patients.^109^

### Meningitis

Meningitis is typically treated with bactericidal agents that easily reach the CSF. However, few studies have assessed the use of bacteriostatic antibacterials, although some can readily penetrate the CNS. The first bacteriostatic antibacterial to show efficient CSF levels and success in the treatment of meningitis was chloramphenicol. In fact, in developing countries, it is often the first-line antibacterial for acute bacterial meningitis due to *Haemophilus influenzae* and *Streptococcus pneumoniae.*^110^ However, due to its toxicity, such as bone marrow suppression and emergence of bacterial resistance, as well as studies showing better results with β-lactams, it is rarely used in developed countries. Nevertheless, data on its success in treating bacterial meningitis highlight that bacteriostatic antibacterials have the potential to result in cure of CNS infections.^1^

There are newer small studies that highlight the use of other bacteriostatic drugs in treating bacterial meningitis. Linezolid is one such drug that has also shown good CSF penetration, with drug levels reaching 30%–70% of its serum levels.^111,112^ A small number of studies have assessed the effectiveness of linezolid in treating meningitis caused by Gram-positive bacteria in combination with other bactericidal antibacterials or as salvage therapy.

A case series of 17 patients from Turkey studied the use of linezolid either as primary therapy or as salvage therapy in the treatment of meningitis by MRSA and methicillin-resistant coagulase-negative staphylococci in post-neurosurgical patients, after failure of first-line treatment with vancomycin and third-generation cephalosporin.^113^ Fifteen of the 17 patients had microbiological clearance of infection by Day 5 (88% microbiological cure rate), and none had a relapse, with only one death due to treatment failure.^113^ Importantly, there were no reported severe haematological, nephrological or hepatological adverse effects due to linezolid.^113^ Another study followed seven patients with penicillin-resistant pneumococcal meningitis who were treated with linezolid, ceftriaxone and dexamethasone. Of the seven patients, one died and one suffered severe sequelae.^114^ Another small study compared treatment of MRSA meningitis with vancomycin or linezolid in 17 patients.^115^ The results showed that 7/9 patients treated with linezolid achieved microbiological cure, compared with 2/8 of those treated with vancomycin (*P* = 0.044), with no severe adverse events occurring in either group.^115^ There has also been a case report of a post-neurosurgical patient who developed VRE meningitis that was successfully treated with IV linezolid, resulting in clinical and microbiological cure.^116^ These studies highlight that linezolid could prove effective in treating Gram-positive meningitis, especially MRSA meningitis, but larger controlled studies are still needed to clearly evaluate its safety and efficacy, especially in comparison with vancomycin.

### Conclusion

The conventional classification of antibacterials as either bactericidal or bacteriostatic based on *in vitro* conditions, lacks clarity in the clinical setting, as indicated by current evidence. Some drugs categorized as ‘bacteriostatic’ may exhibit ‘bactericidal’ effects under specific *in vitro* conditions, and vice versa, considering the complexities observed in clinical contexts and the evolving understanding of antibacterial action. Therefore, predicting whether a particular antibacterial will act as bacteriostatic or bactericidal in an actual patient with a bacterial infection is likely to be highly challenging. Moreover, the drug’s action may be contingent on factors such as bacterial load and interaction with the immune system at the infection site.

Extensive evidence indicates that the effectiveness of bactericidal and bacteriostatic agents is comparable when employed in the treatment of various infections, including severe and complicated SSTIs, community- and hospital-acquired pneumonia, intra-abdominal infections and osteomyelitis. Most studies directly comparing these two categories of antibacterial agents have revealed no discernible disparities in clinical outcomes or mortality. In instances where differences have been observed, the bacteriostatic agent has often demonstrated superiority over its bactericidal counterpart. When bacteriostatic agents were deemed less effective, the likely explanation points to insufficient dosing and/or attainable levels at the infection site, rather than the speed and effectiveness of microbe eradication. Therefore, it is imperative to reconsider the notion that bactericidal antibacterials are inherently more efficacious than bacteriostatic agents. In certain situations, specific bacteriostatic antibacterials such as linezolid and tigecycline may serve as valuable options, particularly in cases of Gram-positive bacteraemia, endocarditis and neutropenic fever, possibly as a form of salvage therapy.
